# Nutritional status of HIV-infected children at Federal University Teaching Hospital, Owerri, Nigeria: A prospective analysis of rural and urban dwellers: Retraction

**DOI:** 10.1097/MD.0000000000039992

**Published:** 2024-10-04

The article, “Nutritional status of HIV-infected children at Federal University Teaching Hospital, Owerri, Nigeria: A prospective analysis of rural and urban dwellers,”^[1]^ which appears in Volume 103, Issue 34 of *Medicine*, is being retracted due to concerns over the omission of coauthors and an ongoing dispute over authorship. As a result, the Publisher no longer has confidence in the actual authorship of this article. The author(s) have been informed of this retraction.
